# Analysis of the risk factors associated with complications following CT-guided percutaneous core needle biopsy of small pulmonary nodules (≤1.5 cm): A single-center retrospective study

**DOI:** 10.1097/MD.0000000000046117

**Published:** 2025-11-21

**Authors:** Jingang Yang, Xiaojun Mo, Fa Wu, Hongmei Yu, Pingping He, Feizhou Du

**Affiliations:** a Department of Radiology, The General Hospital of Western Theater Command, Chengdu, Sichuan Province, China.

**Keywords:** core needle biopsy (CNB), CT-guided, pneumothorax, pulmonary hemorrhage

## Abstract

The aim of this study was to identify the risk factors associated with complications following computed tomography (CT)-guided percutaneous core needle biopsy (PCNB) of small pulmonary nodules (≤1.5 cm). From August 2019 to May 2023, the baseline and procedural technique characteristics of 107 consecutive patients who underwent PCNB for small pulmonary nodules (≤1.5 cm) in our hospital were analyzed retrospectively. The predictors of procedure-related pneumothorax and pulmonary hemorrhage were determined using univariate and multivariate logistic regression analyses. The incidences of pneumothorax and pulmonary hemorrhage were 43% (46/107) and 59.8% (64/107), respectively. Chronic obstructive pulmonary disease (COPD) (OR = 3.453, 95% CI = 1.431–11.532, *P* = .008) and a transpulmonary needle pathway > 3.0 cm (OR = 4.498, 95% CI = 1.269–9.398, *P* = .015) were independent risk factors for pneumothorax. A transpulmonary needle pathway > 3.0 cm (OR = 4.398, 95% CI = 1.350–14.324, *P* = .014) and a needle tract within the lesion > 1.0 cm (OR = 3.017, 95% CI = 1.303–6.986, *P* = .010) were independent risk factors for postprocedure pulmonary hemorrhage. CT-guided PCNB is a safe technique for small pulmonary nodules ≤ 1.5 cm, with acceptable complication rates. Identifying the predictors of pneumothorax and pulmonary hemorrhage postprocedure can help reduce the risk of such complications in future clinical practice.

## 1. Introduction

Lung cancer is the major cause of morbidity and mortality worldwide. Approximately 2.48 million new cases of lung cancer are estimated to be diagnosed in 2022, resulting in 1.81 million deaths, with a discouraging 5-year survival rate of <20%.^[1]^ Early-stage lung cancer typically manifests as solitary pulmonary nodules on chest computed tomography (CT) images. The widespread adoption of high-resolution CT-based lung cancer screening, coupled with advancements in artificial intelligence technology, has significantly increased the detection rate of these pulmonary nodules.

It has been reported^[2]^ that pulmonary nodules measuring 5 to 10 mm have a malignant transformation rate of approximately 1.3%, whereas nodules measuring 10 to 20 mm have a rate of approximately 50%. The management of nodules in the 10 to 20 mm range remains contentious, with biopsy or imaging surveillance decisions informed by evidence-based guidelines.^[3]^ Differentiating benign nodules from malignant nodules using molecular biology assays and imaging techniques, such as fiberoptic bronchoscopy and thoracoscopy, can be challenging. CT-guided percutaneous core needle biopsy (PCNB) offers high diagnostic accuracy for pulmonary nodules.^[4–6]^ Obtaining pathological and molecular data is essential for accurate diagnosis and treatment of lung cancer. Despite its widespread clinical use, this invasive technique is associated with inherent surgical risks.

CT-guided PCNB of pulmonary nodules smaller than 1.5 cm is challenging for operators. To our knowledge, studies on the risk factors associated with the complications of small lesions are still lacking.

Therefore, the aim of this study was to identify the predictors of pneumothorax and pulmonary hemorrhage following biopsy of small pulmonary nodules measuring <1.5 cm to reduce the risk of such postprocedural complications.

## 2. Materials and methods

This retrospective study was approved by the Ethics Committee of Western Theater General Hospital (IRB No. 2021xjsxxm-2003-1). Informed consent was obtained from all patients.

### 2.1. Study population

Data from 107 consecutive patients who underwent CT-guided PCNB of small pulmonary nodules (≤1.5 cm) in our hospital from August 2019 to May 2023 were retrospectively analyzed. The sample included 58 males and 49 females, with a mean age of 55.6 ± 13.4 years (range: 19–79 years).

The inclusion criteria were as follows: small solitary pulmonary nodules (≤1.5 cm); enhanced CT examination within 1 week before CT-guided PCNB; preprocedure assessment confirming no contraindications to interventional procedures and a safe puncture path; signed surgical consent forms.

The exclusion criteria were as follows: pulmonary nodules with a diameter > 1.5 cm; and failed biopsy sampling, incomplete clinical data, or incomplete procedural records.

Contraindications: severe and uncorrectable coagulation dysfunction, emphysema, cardiopulmonary insufficiency, or pulmonary hypertension; large pulmonary bullae obstructing the puncture pathway, vascular malformations, or aneurysms near the lesion; suspected pulmonary echinococcosis, mental disorders, or frailty that impedes cooperation or tolerance to surgery.

### 2.2. Equipment and materials

All biopsies were guided by a 64-detector spiral CT scanner (Somatom Sensation 64, Siemens, Munich, Germany), a semiautomatic core needle (Curaway, Zhejiang, China), a custom-made radiopaque grid, and resuscitation medicines.

### 2.3. PCNB procedure

All procedures were performed by 2 interventional radiologists with 5 or 10 years of experience. Upon selecting the puncture route, care was taken to avoid the ribs and scapulas, bypass fissures, avoid pulmonary alveoli, and avoid crucial vasculature. Patients were advised to maintain calm breathing, refrain from talking, and avoid severe coughing during the procedure.

The parameters for the chest CT scan included a tube voltage of 120 kV and a tube current of 120 mAs, with a 5 mm section thickness. The primary objective of the CT scan was to locate the puncture site and determine the trajectory of needle insertion. Following sterilization of the puncture site and application of a sterile drape, local anesthesia was administered with 2% lidocaine. A 17-gauge introducer needle was used following the surgical protocol. The direction, angle, and depth of the puncture were determined using CT images. Upon safely reaching the periphery of the lesion, the inner core of the coaxial needle was extracted, and an 18-gauge semiautomatic cutting needle was introduced to procure 1 to 2 tissue samples. The tissue samples were preserved in formaldehyde solution and sent for histopathological analysis.

### 2.4. Data collection and definitions

In this study, we documented the clinical characteristics, imaging features of pulmonary nodules, and technical factors related to percutaneous biopsy, including nodule enhancement patterns, cavity and necrosis status, pleura–needle angle, transpulmonary needle pathway length, and needle tract length within the lesions. The lung nodules were classified into 2 categories based on the location of the lesion: central pulmonary nodules were defined as lesions located < 2 cm from the hilum, whereas nodules in other locations were defined as peripheral nodules. We defined abnormal feeding arteries with a diameter exceeding 4 mm within or at the edge of the lesions as enlarged arteries.

Pneumothorax, pulmonary hemorrhage, hemoptysis, pleural effusion, and other rare complications after PCNB were also recorded. Pneumothorax was graded according to the degree of compression (grade I: <30%, grade II: 30–60%, grade III: >60%), and the time of the appearance of pneumothorax and whether it was resolved by closed thoracic drainage were recorded. Hemorrhage was classified into 3 grades according to the extent of bleeding around the puncture tract (grade I: <2 cm; grade II: 2–5 cm; grade III: >5 cm).

### 2.5. Statistical analysis

All the data were analyzed and processed using Statistical Program for Social Sciences 21.0 software (SPSS Inc., Chicago). Quantitative data are presented as means ± SDs (x ± s), whereas qualitative data are presented as relative numbers (n) and percentages (%). Continuous variables between the groups, assuming a normal distribution, were compared using independent t tests, and categorical variables were compared using either the chi-square test or Fisher exact test. The risk factors for postprocedural complications were assessed using univariate and multivariate logistic regression analyses. Statistical significance was set at *P* < .05.

## 3. Results

### 3.1. Complications

**Figure 1.**
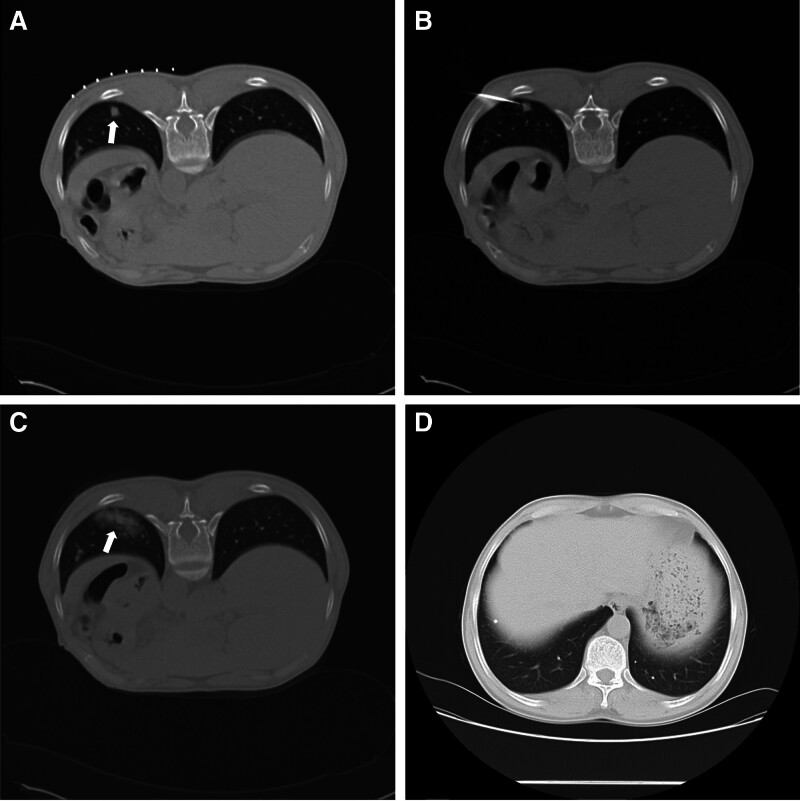
(A) A 57-yr-old male patient exhibited an 11 mm inflammatory nodule in the left lower lobe (white arrow). (B) CT-guided PCNB was performed using an 18-gauge cutting needle. (C) Transpulmonary needle pathway with alveolar hemorrhage after PCNB (white arrow). (D) 6 mo later, the pulmonary nodules had disappeared. CT = computed tomography, PCNB = percutaneous core needle biopsy.

**Figure 2.**
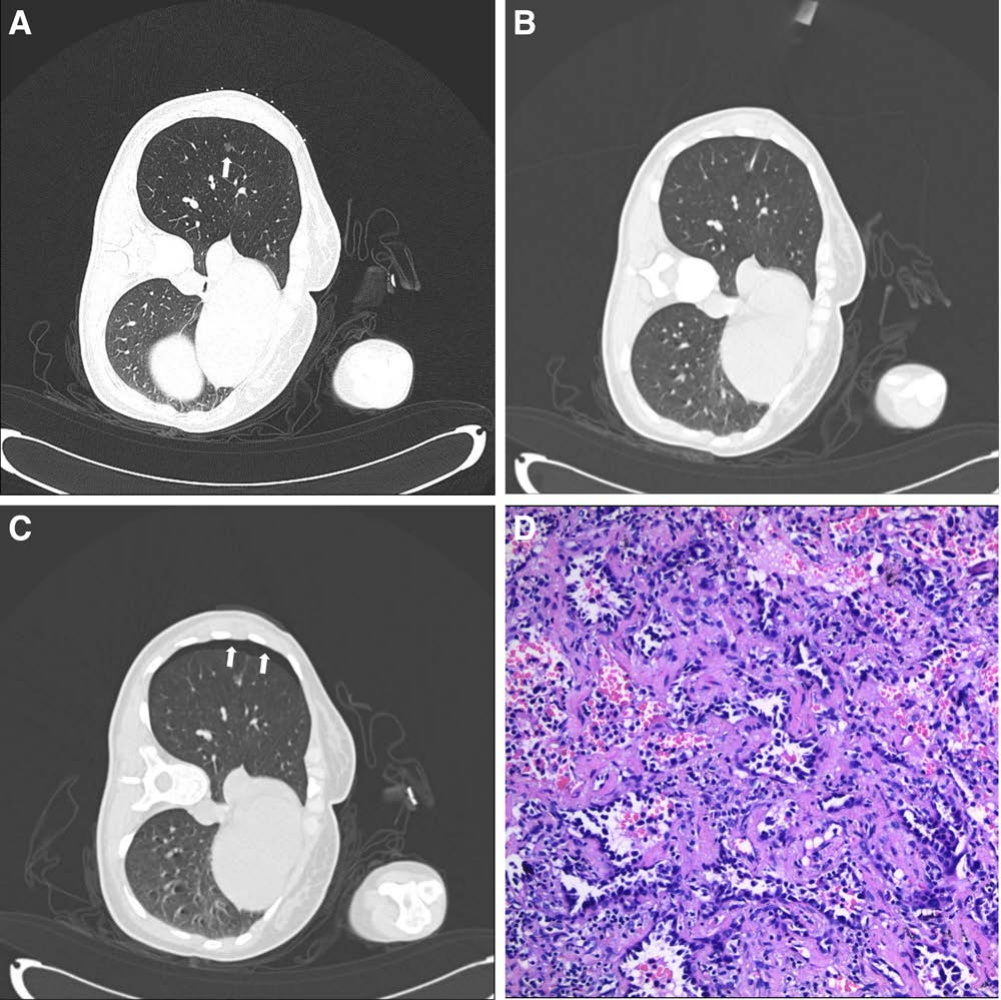
(A) A 37-yr-old female patient presented with an 8 mm nodule in the right lower lobe (white arrow). (B) CT-guided PCNB was performed using an 18-gauge cutting needle. (C) Mild pneumothorax occurred immediately after PCNB (white arrow). (D) Postoperative pathology (hematoxylin and eosin staining, ×100): invasive adenocarcinoma. CT = computed tomography, PCNB = percutaneous core needle biopsy.

A total of 107 patients were included in this study. All patients underwent successful PCNB procedures. Hemorrhage occurred in 64 patients (59.8%) following sampling, with 60 patients (56.1%) classified as having grade I and II hemorrhages that did not require treatment (Fig. 1). Pneumothorax was observed in 46 patients (43%) after PCNB, including 44 patients (41.1%) with grade I pneumothorax (Fig. 2). Only 2 patients (1.9%) underwent chest tube drainage for severe pneumothorax. Additionally, there were 4 cases of mild hemoptysis (3.7%), 2 cases of hemothorax (1.8%), and 1 case of mild intramural hematoma of the aorta (0.9%). Notably, no severe complications, such as air embolism or mortality, were reported.

### 3.2. Univariate analysis of patients with pneumothorax and pulmonary hemorrhage

A total of 107 patients with small pulmonary nodules (≤1.5 cm) underwent successful biopsies. The patients were categorized into groups on the basis of the occurrence of pneumothorax or hemorrhage. Univariate analysis of the clinical data, morphological characteristics of the lung nodules, and technique-related variables was performed between the groups.

Univariate analysis revealed that the occurrence of pneumothorax was significantly associated with chronic obstructive pulmonary disease (COPD) (*P* = .015), the puncture position (*P* = .047), the transpulmonary needle pathway (*P* = .049), and the number of pleural passes (*P* = .010). Conversely, no significant differences were observed in age, smoking history, location of pulmonary nodules, lesion–pleura distance, density of pulmonary nodules, pleural-needle angle, procedure duration, number of cuttings, or sampling length between the groups (*P* > .05) (Table 1).

**Table 1:** Univariate analysis of the risk factors for pulmonary hemorrhage and pneumothorax (n = 107).

Variables	Pneumothorax, n (%)			Hemorrhage, n (%)		
	No	Yes	*P* value	No	Yes	*P* value
Sex						
Male	34 (31.8)	24 (22.4)	.714	18 (16.8)	40 (37.4)	.036
Female	27 (25.2)	22 (20.6)		25 (23.4)	24 (22.4)	
Age	53.3 ± 13.1	58.7 ± 13.4	.115	54.2 ± 13.4	56.6 ± 13.4	.115
≤60 yr	41 (38.3)	24 (22.4)		28 (26.2)	38 (35.5)	
>60 yr	20 (18.7)	22 (20.6)		15 (14)	26 (24.3)	
Smoking	15 (14)	18 (16.8)	.107	15 (14)	25 (23.4)	.661
Hypertension	4 (3.7)	6 (5.6)	.420	5 (4.7)	12 (11.2)	.323
COPD	8 (7.5)	15 (14)	.015	4 (3.7)	10 (9.3)	.342
Nodule size	1.2 ± 0.3	1.2 ± 0.2	.731	1.1 ± 0.3	1.2 ± 0.2	.005
<1.0 cm	18 (16.8)	15 (14)		17 (15.9)	10 (9.3)	
1.0–1.5 cm	43 (40.2)	31 (28.9)		26 (24.3)	54 (50.5)	
Location						
Central	4 (3.7)	0	.209	2 (1.9)	2 (1.9)	.683
Peripheral	57 (53.2)	46 (42.9)		41 (38.3)	62 (57.9)	
Lesion–pleura distance	1.1 ± 1.1	1.3 ± 1.1	.148	1.1 ± 1.0	1.2 ± 1.2	.743
<2 cm	53 (49.5)	35 (32.7)		36 (33.6)	52 (48.5)	
≥2 cm	8 (7.5)	11 (10.3)		7 (6.5)	12 (11.2)	
Nodule density						
GGO	15 (14)	13 (12.1)	.528	18 (16.8)	10 (9.3)	.010
PSN	8 (7.5)	3 (2.8)		3 (2.8)	8 (7.5)	
SN	38 (35.5)	30 (28)		22 (20.5)	46 (42.9)	
Necrosis	0	1 (0.9)	.430	0	1 (0.9)	1.000
Cavity	7 (6.5)	3 (2.8)	.592	3 (2.8)	7 (6.5)	.725
Enlarged arteries	6 (5.6)	3 (2.8)	.795	3 (2.8)	11 (10.3)	.125
Patient position						
Supine	25 (23.4)	13 (12.1)	.047	17 (15.9)	21 (19.6)	.764
Prone	28 (26.1)	18 (16.8)		17 (15.9)	29 (27.1)	
Lateral	8 (7.5)	15 (14)		9 (8.4)	14 (13.1)	
Pleural-needle angle						
≤45°	20 (18.7)	10 (9.3)	.208	11 (10.3)	19 (17.8)	.643
45–90°	41 (38.3)	36 (33.6)		32 (29.9)	45 (42.1)	
Number of pleural passes						
1 time	28 (26.2)	10 (9.3)	.010	19 (17.8)	19 (17.8)	.124
≥2 times	33 (30.8)	36 (33.6)		24 (22.4)	45 (42.1)	
Transpulmonary needle pathway	2.2 ± 1.4	2.3 ± 1.6	.050	1.7 ± 1.1	2.6 ± 1.5	.012
≤3.0 cm	51 (47.7)	31 (28.9)		38 (35.5)	43 (40.2)	
>3.0 cm	10 (9.3)	15 (14)		5 (4.7)	21 (19.6)	
Needle tract within the lesion	1.3 ± 0.5	1.2 ± 0.5	.411	1.1 ± 0.4	1.4 ± 0.5	.004
≤1.0 cm	26 (24.3)	16 (14.9)		24 (22.4)	18 (16.8)	
>1.0 cm	35 (32.7)	30 (28)		19 (17.8)	46 (42.9)	
Sampling number						
1 time	42 (39.2)	32 (29.9)	.937	25 (23.4)	49 (45.8)	.043
≥2 times	19 (17.8)	14 (13.1)		18 (16.8)	15 (14)	
Procedure time	8.9 ± 4.3	11.4 ± 5.8	.135	8.8 ± 4.7	10.8 ± 5.3	.122
<10 min	38 (35.5)	22 (20.6)		28 (26.2)	32 (29.9)	
≥10 min	23 (21.5)	24 (22.4)		15 (14)	32 (29.9)	
Sample length	0.8 ± 0.4	0.8 ± 0.3	.716	0.7 ± 0.3	0.9 ± 0.4	.282
<1.0 cm	35 (32.7)	28 (26.2)		28 (26.2)	35 (32.7)	
1.0–2.0 cm	26 (24.3)	18 (16.8)		15 (14)	29 (27.1)	
Operator experience						
5 years	26	25	.229	23	28	.323
10 years	35	21		20	36	

COPD = chronic obstructive pulmonary disease, GGO = ground-glass nodules, PSN = partial solid nodules, SN = solid nodules.

Univariate analysis revealed that the occurrence of hemorrhage was significantly associated with sex (*P* = .036), the diameter of the pulmonary nodule (*P* = .010), the density of the pulmonary nodules (*P* = .010), the transpulmonary needle pathway (*P* = .01), the length of the needle tract within the lesion (*P* = .004), and the number of samples (*P* < .01). Other variables, such as age, COPD, location, necrosis and cavities, patient position during the procedure, pleural-needle angle, number of pleural passes, procedure duration, and sampling location, were not significantly different between the groups. (*P* > .05) (Table 1).

### 3.3. Multivariate logistic regression analyses of patients with pneumothorax and pulmonary hemorrhage

**Figure 3.**
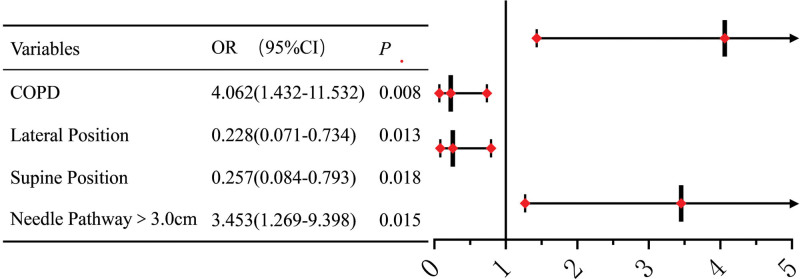
Logistic regression visual forest map of independent risk factors for pneumothorax. COPD = chronic obstructive pulmonary disease, CI = confidence interval, OR = odds ratio.

The multivariable logistic regression results are shown in Figure 3. COPD (OR = 3.453, 95% CI = 1.431–11.532, *P* = .008) and a transpulmonary needle pathway in the lung > 3.0 cm (OR = 4.498, 95% CI = 1.269–9.398, *P* = .015) were identified as independent risk factors for pneumothorax after the procedure in patients with pulmonary nodules ≤ 1.5 cm. Additionally, the lateral puncture position (OR = 0.228, 95% CI = 0.071–0.734; *P* = .013) and supine position (OR = 0.257, 95% CI = 0.084–0.793; *P* = .018) were protective factors against pneumothorax.

**Figure 4.**
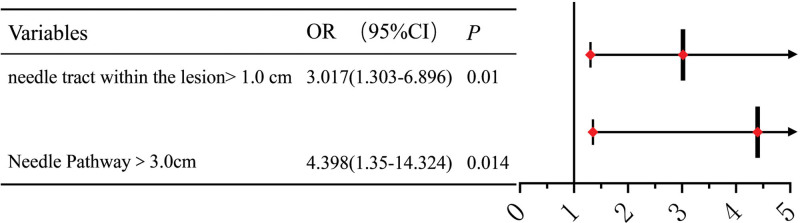
Logistic regression visual forest map of independent risk factors for hemorrhage.

A transpulmonary needle pathway in the lung > 3.0 cm (OR = 4.398, 95% CI = 1.350–14.324, *P* = .014) and a needle tract within the lesion > 1.0 cm (OR = 3.017, 95% CI = 1.303–6.986, *P* = .010) were identified as independent risk factors for postprocedure hemorrhage (Fig. 4).

CI = confidence interval, OR = odds ratio.

## 4. Discussion

CT-guided PCNB is commonly used to identify pulmonary nodules because of its high diagnostic accuracy, minimal invasiveness, and repeatability.^[7]^ Primary postprocedural complications typically involve mild pneumothorax and pulmonary hemorrhage. Rare but severe complications include hemothorax, needle tract seeding, air embolism, and mortality.^[8]^ The clinical emphasis has shifted toward reducing complications associated with puncturing small pulmonary lesions rather than solely enhancing diagnostic accuracy.

Pneumothorax was the most frequent complication following CT-guided PCNB of small pulmonary nodules measuring <1.5 cm in size, with an incidence of 43% (46/107). Previous studies^[9–11]^ reported pneumothorax rates ranging from 16 to 45%, with a rate of pneumothorax requiring chest drain insertion ranging from 1.8 to 15%. In a meta-analysis involving 23,104 patients, Huo et al^[12]^ reported an overall pneumothorax incidence of approximately 25.9%. Although a higher incidence of pneumothorax was observed in the present study, 44 patients (41.1%) experienced minor pneumothorax that resolved spontaneously and did not prolong hospitalization, and only 2 patients (1.9%) required chest drain insertion, which was a lower rate than that reported in previous studies.^[13]^

In this study, we identified COPD as an independent risk factor for pneumothorax, which is consistent with the results of previous studies.^[4,14,15]^ Patients with COPD exhibit lung degeneration, fibrosis, emphysema, and pulmonary bullae. Owing to the susceptibility of lung tissue to damage caused by puncture needles, gas may enter the pleural cavity, leading to pneumothorax. To reduce the occurrence of pneumothorax in patients with COPD, it is advisable to choose the shortest needle path, avoid pulmonary bullae and interlobar fissures, and ensure accurate and rapid pleural penetration. These measures are specifically intended to reduce the frequency of pleural entry and exit, as well as the duration of the procedure.

Compared with the prone position, the supine and lateral positions were identified as protective factors against pneumothorax. The supine position was associated with comfort and cooperation, whereas the prone position was associated with less comfort and cooperation because of the thickness of the back muscles. Although the latter may enhance puncture needle stability, it also complicates needle adjustment. Furthermore, increased movement of the posterior lung during respiration in the prone position increases the risk of needle-induced damage to the pleural and lung tissues, consequently increasing the likelihood of pneumothorax. Although these findings contradict those reported by Li et al,^[16]^ Huo et al^[12]^ reported a significant reduction in the risk of pneumothorax in the lateral position. Discrepancies in study outcomes may be attributed to variations in operator expertise and patient compliance.

Pulmonary hemorrhage is another common complication of CT-guided PCNB. A meta-analysis^[11]^ revealed wide variation in the incidence of pulmonary hemorrhage, ranging from 3.3 to 54.5%. This variability may be attributed to several factors, including the use of the coaxial technique, needle size, nodule size, operator expertise, and potential selection bias in study subjects. In the present study, the incidence of pulmonary hemorrhage was 59.8% (64/107), with 56.1% (60/107) of patients exhibiting grade I hemorrhage, and severe pulmonary hemorrhage observed in 3.7% (4/107) of patients. Furthermore, hemoptysis was observed in 4 patients with bronchiole injuries during the procedure and effectively managed with intravenous hemostatic agents, successfully avoiding any life-threatening complications.

A longer transpulmonary needle pathway is recognized as a significant independent risk factor for pulmonary hemorrhage and pneumothorax.^[14,17,18]^ Consistent with previous findings, our study revealed a 3.4-fold increase in pneumothorax and a 4.3-fold increase in pulmonary hemorrhage when the transpulmonary needle pathway was > 3.0 cm. Lengthening the needle trajectory within the lung inevitably increases the likelihood of damage to bronchioles and associated arterioles. However, the association between the transpulmonary needle pathway and pneumothorax remains a topic of debate among researchers.^[19]^ Li et al^[20]^ reported that an increased distance between nodules and the pleura was closely linked to pulmonary hemorrhage, whereas Andrade et al^[5]^ identified a pleura-to-lesion distance >3.0 cm as a risk factor for pneumothorax. Given that small lesions are often situated deep within the lung, away from the pleura, minimizing the length of the needle path is essential for mitigating these risks. In particular, for subcentimeter solid and ground-glass nodules, extending the needle path increases the complexity of the biopsy procedure, making it challenging to achieve accurate placement in a single attempt. Consequently, this may lead to prolonged puncture procedures and needle adjustments, exacerbating damage to lung tissue and small vessels. These complications increase the risk of pulmonary hemorrhage and pneumothorax. In particular, ground-glass nodules exhibit weaker natural retraction forces owing to their growth pattern along the alveolar walls than solid nodules do. Consequently, damaged microvessels are less effectively compressed by lung tissue retraction after incision, resulting in a higher hemorrhage rate in ground-glass nodules than in solid nodules. To prevent pneumothorax when dealing with subpleural pulmonary nodules, it is advisable to use a needle with a small needle-pleural angle, extend the needle path appropriately to ensure needle stability, and prevent dislodgement caused by respiratory movements.

To our knowledge, reports regarding the needle length within the lesion are limited. Multivariate logistic regression analysis revealed that a needle length > 1.0 cm was an independent risk factor for pulmonary hemorrhage. Although an increased intralesional needle length improves tissue sampling adequacy, it also increases the risk of tissue damage. A previous study^[21]^ highlighted a significant association between subsolid nodules and higher-grade hemorrhage, indicating that subsolid lesions, which are less dense, offer less tamponade in bleeding scenarios. We observed that for small pulmonary nodules (<1.0 cm), particularly ground-glass and subsolid nodules, extending the cutting needle within the lesion may result in the needle tip traversing the nodule into normal lung tissue, making bleeding more likely when normal lung tissue is cut than when the lesion itself is cut. Therefore, before the puncture needle is advanced to the target site and the cutting needle is deployed for sampling, local slice scanning should be routinely conducted to measure and estimate the cutting needle length accurately, thereby minimizing the risk of unnecessary damage to normal lung tissue while ensuring adequate sampling.

This study is significantly advantageous in that it presents the findings of regression analyses of a comprehensive range of predictive factors, such as patient clinical data, imaging features of lung nodules, and procedural indicators, potentially linked to complications after biopsy of small-diameter lesions. These findings indicated that sex, lung nodule diameter, nodule characteristics, and biopsy attempts were significantly correlated with pulmonary hemorrhage, albeit not independent risk factors. Furthermore, the frequency of needle adjustments was significantly associated with pneumothorax; however, the multivariate analysis revealed that it was not an independent risk factor for pneumothorax, contrary to prior research.^[19,22]^ This analysis may be attributed to the small sample size and potential subject selection bias. In this study, the pulmonary nodules were generally small, with a low likelihood of necrosis and cavity formation. Additionally, most of the pulmonary nodules that were examined were superficial, with a pleura–lesion distance of <2.0 cm in 82.2% of the patients (88/107). Most lesions are located under the pleura, thereby significantly simplifying lesion localization and the procedure itself. No statistically significant associations were detected between the key procedural parameters (operation duration and needle insertion angle) and complications. Discrepancies exist among different studies on this matter.^[14,23,24]^ PCNB is a multifaceted procedure that is influenced by variables such as operator expertise and technique. Therefore, increasing the sample size is necessary to systematically investigate potential risk factors for procedure-related complications.

This study has several limitations. First, it was a single-center retrospective study with a small sample size, potentially introducing selection bias. Second, incomplete baseline data, such as lung function tests and pulmonary hypertension assessments, hindered a comprehensive analysis of the possible factors associated with complications. Furthermore, rare complications such as hemoptysis were not subjected to a regression analysis for risk factors. Third, the PCNB procedures were conducted by 2 interventional attending physicians with varying levels of experience, which may be a potential confounding factor for complications. Future research should include prospective, multicenter, large-sample randomized controlled trials to validate these findings, particularly in the context of small lung lesions.

In conclusion, CT-guided PCNB is a safe procedure for small pulmonary nodules (≤1.5 cm), with mild complications. Optimal planning of the puncture trajectory, minimizing the length of the transpulmonary needle pathway, and reducing the length of the cutting needle in the lesion while ensuring adequate sampling can help reduce complications.

## Author contributions

**Data curation:** Xiaojun Mo.

**Formal analysis:** Fa Wu.

**Methodology:** Pingping He.

**Supervision:** Hongmei Yu.

**Writing – original draft:** Jingang Yang.

**Writing – review & editing:** Feizhou Du.
